# Supplementary material for: Statistical analysis supports pervasive RNA subcellular localization and alternative 3' UTR regulation
Source: eLife. 2024 Dec 19;12:RP87517. doi: 10.7554/eLife.87517 (PMC11658768; doi:10.7554/eLife.87517)
Supplement: Supplementary file 1. — Genes are defined as significant if they are observed to be significant in at least cell-type in any replicate. Opposite-effect genes are those observed to have at least one significantly positive Subcellular Patterning Ranked Analysis With Labels (SPRAWL) gene/cell-type score, and one significantly negative SPRAWL gene/cell-type score. [file elife-87517-supp1.docx]

## Supplemental Table 1

| **Experiment** | **Metric** | **Unique genes** | **Significant genes** | **Bidirectional genes** |
| --- | --- | --- | --- | --- |
| Vizgen Brainmap | Peripheral | 589 | 224 | 3 |
| Vizgen Brainmap | Central | 589 | 208 | 2 |
| Vizgen Brainmap | Radial | 370 | 77 | 0 |
| Vizgen Brainmap | Punctate | 370 | 138 | 3 |
| Vizgen Liver | Peripheral | 385 | 112 | 215 |
| Vizgen Liver | Central | 385 | 112 | 202 |
| Vizgen Liver | Radial | 385 | 134 | 64 |
| Vizgen Liver | Punctate | 385 | 118 | 64 |
| BICCN MERFISH | Peripheral | 252 | 251 | 92 |
| BICCN MERFISH | Central | 252 | 249 | 96 |
| BICCN MERFISH | Radial | 251 | 216 | 2 |
| BICCN MERFISH | Punctate | 251 | 236 | 10 |
| SeqFISH+ | Peripheral | 9805 | 155 | 0 |
| SeqFISH+ | Central | 9805 | 113 | 1 |
| SeqFISH+ | Radial | 2423 | 907 | 131 |
| SeqFISH+ | Punctate | 2423 | 815 | 83 |
| CZB Kidney | Peripheral | 307 | 273 | 44 |
| CZB Kidney | Central | 307 | 270 | 58 |
| CZB Kidney | Radial | 272 | 250 | 83 |
| CZB Kidney | Punctate | 272 | 250 | 68 |
| CZB Liver | Peripheral | 305 | 249 | 44 |
| CZB Liver | Central | 305 | 240 | 43 |
| CZB Liver | Radial | 162 | 136 | 67 |
| CZB Liver | Punctate | 162 | 136 | 28 |
